# Association of Brain Cancer With Risk of Suicide

**DOI:** 10.1001/jamanetworkopen.2020.3862

**Published:** 2020-05-01

**Authors:** Anas M. Saad, Abdelmagid M. Elmatboly, Mohamed M. Gad, Muneer J. Al-Husseini, Khalid A. Jazieh, Muayad A. Alzuabi, Ahmad Samir Alfaar

**Affiliations:** 1 Heart and Vascular Institute, Cleveland Clinic Foundation, Cleveland, Ohio; 2 Faculty of Medicine, Al-Azhar University, Cairo, Egypt; 3 Department of Internal Medicine, Cleveland Clinic Foundation, Cleveland, Ohio; 4 Gillings School of Global Public Health, University of North Carolina, Chapel Hill; 5 Department of Medicine, Ascension St John Hospital, Detroit, Michigan; 6 Department of Neuroscience, Medical University of South Carolina, Charleston; 7 Experimental Ophthalmology, Charité-Universitatsmedizin Berlin, Berlin, Germany; 8 Ophthalmology Department, Faculty of Medicine, Leipzig University, Leipzig, Germany

## Abstract

This cohort study examines outcomes of patients with brain cancer to provide demographic details on the increase in suicide rate after brain cancer diagnosis.

## Introduction

It was estimated that there would be 23 820 new cases of central nervous system tumors in the US in 2019, along with 17 760 deaths.^1^ In our previous work,^2^ we found that compared with the general population, suicide rates within the first year of cancer diagnosis are significantly higher, particularly in cancers with poor prognosis. This new work aims to investigate the increase in suicide rate associated with diagnosis with brain cancer.

## Methods

Data for patients diagnosed with brain cancers between 2000 and 2016 were collected from the National Cancer Institute’s Surveillance, Epidemiology, and End Results (SEER) Program registries,^3^ accessed and analyzed in June 2019. An event was defined as death due to suicide following a brain cancer diagnosis. The observed to expected (O/E) event ratio was calculated to determine the change in suicide risk following diagnosis in comparison with the general population, in which expected number of events is the number of people estimated to die of the same cause in a demographically similar general population within the same period. Mortality data for the general US population were collected by the National Center for Health Statistics.^3^ All statistical tests were 2-sided, and *P* < .05 was used as a cutoff for statistical significance. This study followed the Strengthening the Reporting of Observational Studies in Epidemiology (STROBE) reporting guidelines and was exempted from institutional review board approval because the SEER data are anonymized and considered non–human participant research.

### Results

A total of 87 785 patients with brain cancer diagnosed between January 2000 and December 2016 were included in the analysis, of whom 29 patients (0.03%) died by suicide and 33 993 (38.7%) died of cancer and other causes within the first year of their diagnosis. Most patients who died of suicide were men (27 [93.1%]), were older than 44 years (24 [82.8%]), were white (26 [89.9%]), and had a glioblastoma (18 [62.1%]). The risk of suicide increased significantly within the first year following a diagnosis of brain cancer, with an O/E of 3.05 (95% CI, 2.04-4.37). Although 48 908 diagnoses (55.2%) were in men, 27 patients (93.1%) who committed suicide were men, with a significant increase in risk to an O/E of 3.38 (95% CI, 2.23-4.92). The increase in suicide risk was highest among patients older than 64 years, who had an O/E of 5.04 (95% CI, 2.60-8.80). White patients also showed an increased risk, with an O/E of 2.91 (95% CI, 1.90-4.26) (Table).

**Table.** Baseline Characteristics and Standard Mortality Rates for Patients with Brain Tumor Patients Who Died of Suicide

Characteristic	Within the first year				After more than a year			
	Total patients, No. (%)	Observed, No. (%)	Expected, No.	O/E (95% CI)	Total patients, No. (%)	Observed, No. (%)	Expected, No.	O/E (95% CI)
Total	87 785 (100)	29 (100)	9.52	3.05 (2.04-4.37)^a^	48 185 (100)	35 (100)	29.79	1.17 (0.82-1.63)
Sex								
Men	48 908 (55.7)	27 (93.1)	7.98	3.38 (2.23-4.92)^a^	27 035 (56.1)	29 (82.9)	24.08	1.20 (0.81-1.73)
Women	38 877 (44.3)	2 (6.9)	1.54	1.30 (0.16-4.68)	21 150 (43.9)	6 (14.1)	5.71	1.05 (0.39-2.29)
Age at diagnosis, y								
<18	11 657 (13.3)	0	0.18	0 (0-19.98)	9420 (19.5)	3 (8.6)	3.7	0.81 (0.17-2.37)
18-44	17 921 (20.4)	5 (17.2)	2.57	1.94 (0.63-4.53)	14 402 (29.9)	17 (48.6)	13.72	1.24 (0.72-1.98)
45-64	30 059 (34.2)	12 (41.4)	4.38	2.74 (1.42-4.79)^a^	17 140 (35.6)	10 (28.6)	9.87	1.01 (0.49-1.86)
>64	28 148 (32.1)	12 (41.4)	2.38	5.04 (2.60-8.80)^a^	7223 (15.0)	5 (14.2)	2.49	2.01 (0.65-4.69)
Race								
White	75 616 (86.1)	26 (89.8)	8.94	2.91 (1.90-4.26)^a^	40 967 (85.0)	30 (85.5)	27.72	1.08 (0.73-1.55)
Black	6511 (7.4)	1 (3.4)	0.26	3.89 (0.10-21.66)	3801 (7.9)	1 (2.9)	0.97	1.03 (0.03-5.71)
Asian or Pacific Islander	5258 (6.0)	2 (6.8)	0.3	6.71 (0.81-24.25)	3175 (6.6)	3 (8.7)	1.01	2.97 (0.61-8.68)
American Indian or Alaska Native	400 (0.5)	0	0.02	0 (0-161.85)	242 (0.5)	1 (2.9)	0.09	11.32 (0.29-63.06)
Marital status								
Married	44 040 (50.2)	17 (58.8)	6	2.83 (1.65-4.54)^a^	23 283 (48.3)	12 (34.3)	15.54	0.77 (0.40-1.35)
Single	26 487 (30.2)	4 (13.7)	2.06	1.94 (0.53-4.96)	18 493 (38.4)	20 (57.2)	10.76	1.86 (1.14-2.87)
Separated	710 (0.8)	0	0.08	0 (0-45.89)	368 (0.8)	1 (2.9)	0.25	4.08 (0.10-22.73)
Divorced	5966 (6.8)	2 (6.8)	0.64	3.13 (0.38-11.31)	2783 (5.8)	1 (2.9)	1.67	0.60 (0.02-3.34)
Widowed	7278 (8.3)	1 (3.4)	0.34	2.91 (0.07-16.24)	1583 (3.3)	1 (2.9)	0.42	2.37 (0.06-13.20)
Unknown	3185 (3.6)	5 (17.3)	NA	NA	1609 (3.3)	0	NA	NA
Brain tumor histology								
Glioblastoma	39 248 (44.7)	18 (62.1)	4.5	4 (2.37-6.32)^a^	14 609 (30.3)	7 (20)	3.8	1.84 (0.74-3.79)
Other brain histologies	48 537 (55.3)	11 (37.9)	5.02	2.19 (1.09-3.92)^a^	33 576 (69.9)	28 (80)	25.99	1.08 (0.72-1.56)

Abbreviation: NA, not applicable; O/E, observed to expected ratio.

^a^ *P* < .05.

## Discussion

The results of this study show that diagnosis with brain cancer is associated with an increased risk of suicide within the first year following diagnosis. This increase was not uniform across all subgroups, with male patients with glioblastoma multiform (GBM) having the highest increase in risk. Diagnosis of GBM may result in profound consequences on patients’ quality of life, not only because it is an aggressive terminal illness but also owing to the nature and sensitive location of these malignant neoplasms. Varying tumor factors, such as lesion size and location within the central nervous system, may affect neurocognitive functions^4^ and result in debilitations that can impair quality of life. For example, loss of motor function or aphasia can significantly affect patients’ ability to work or care for themselves. Radiation or surgery may result in more local damage and can worsen neurologic manifestations. The implications of these impairments include early retirement or unemployment, loss of independence, feeling like a burden to caregivers, and instability of interpersonal relationships, all of which may also explain the higher rates of depression and suicide among older male patients with brain cancer.^5^^,^^6^ Our study has several limitations, including the potential for bias given the retrospective nature of the analysis, along with the absence of data on anxiety and depression after a cancer diagnosis.

In this study, patients diagnosed with brain cancers showed a higher incidence of suicide within the first year following their diagnosis compared with the general population. Integrating comprehensive active psychiatric care plans into the management protocols of these patients could improve their quality of life and reduce suicide rates.
